# Environmental radon exposure and breast cancer risk in the Nurses’ Health Study II

**DOI:** 10.1186/s12940-017-0305-6

**Published:** 2017-09-07

**Authors:** Trang VoPham, Natalie DuPré, Rulla M. Tamimi, Peter James, Kimberly A. Bertrand, Veronica Vieira, Francine Laden, Jaime E. Hart

**Affiliations:** 1 Department of Epidemiology, Harvard T.H. Chan School of Public Health, Landmark Center 3rd Floor West (HSPH/BWH), 401 Park Drive, Boston, MA 02215 USA; 2 Channing Division of Network Medicine, Department of Medicine, Brigham and Women’s Hospital and Harvard Medical School, Boston, MA USA; 3 Exposure, Epidemiology, and Risk Program, Department of Environmental Health, Harvard T.H. Chan School of Public Health, Boston, MA USA; 4 Slone Epidemiology Center at Boston University, Boston, MA USA; 5 Program in Public Health, University of California, Irvine, CA USA

**Keywords:** Radon, Ionizing radiation, Breast cancer

## Abstract

**Background:**

Radon and its decay products, a source of ionizing radiation, are primarily inhaled and can deliver a radiation dose to breast tissue, where they may continue to decay and emit DNA damage-inducing particles. Few studies have examined the relationship between radon and breast cancer.

**Methods:**

The Nurses’ Health Study II (NHSII) includes U.S. female registered nurses who completed biennial questionnaires since 1989. Self-reported breast cancer was confirmed from medical records. County-level radon exposures were linked with geocoded residential addresses updated throughout follow-up. Time-varying Cox regression models adjusted for established breast cancer risk factors were used to calculate hazard ratios (HRs) and 95% confidence intervals (CIs).

**Results:**

From 1989 to 2013, 3966 invasive breast cancer cases occurred among 112,639 participants. Increasing radon exposure was not associated with breast cancer risk overall (adjusted HR comparing highest to lowest quintile = 1.06, 95% CI: 0.94, 1.21, p for trend = 0.30). However, women in the highest quintile of exposure (≥74.9 Bq/m^3^) had a suggested elevated risk of ER−/PR- breast cancer compared to women in the lowest quintile (<27.0 Bq/m^3^) (adjusted HR = 1.38, 95% CI: 0.97, 1.96, p for trend = 0.05). No association was observed for ER+/PR+ breast cancer.

**Conclusions:**

Although we did not find an association between radon exposure and risk of overall or ER+/PR+ breast cancer, we observed a suggestive association with risk of ER−/PR- breast cancer.

**Electronic supplementary material:**

The online version of this article (10.1186/s12940-017-0305-6) contains supplementary material, which is available to authorized users.

## Background

Breast cancer is the most commonly occurring type of cancer, excluding non-melanoma skin cancer, and the leading cause of cancer-related death among women worldwide [1]. International and national geographic variation in breast cancer incidence suggests that environmental exposures may play a role in breast carcinogenesis [2]. Ionizing radiation is a type of electromagnetic radiation that is able to break chemical bonds in molecules such as DNA [3]. Ionizing radiation from diagnostic/therapeutic sources and atomic bombs is an established breast cancer risk factor [4–9]. However, the relationship between radon, an ionizing radioactive gas and International Agency for Research on Cancer (IARC) Group 1 human carcinogen [10], and breast cancer has not been well characterized. Radon is a naturally occurring radioactive gas forming from the decay of uranium and thorium (e.g., uranium-238 and thorium-232) [11], found in air, soil, rocks, and water [12]. The primary source of indoor radon is from soil gas entering homes through foundation cracks via pressure-driven flow [13]. Groundwater may also contain high concentrations of radon due to uranium-rich rocks and soils, which can be outgassed to indoor air from washing and cooking. Approximately 6% of U.S. homes have radon levels above the Environmental Protection Agency (EPA) action level of 148 Bq/m^3^ at which remediation is recommended [14]. Radon decays into its decay products (e.g., polonium-218), both of which can enter the human body primarily through inhalation, emitting radiation in the form of alpha particles, beta particles, and/or gamma rays. This radiation is predicted to deliver radiation doses to various organs and tissues including the lung and breast, which can cause DNA damage and generate oxidative stress [13, 15].

Radon and radon decay products have been predicted to deliver radiation doses to breast tissue [15, 16]. Although most inhaled radon gas is subsequently exhaled, the majority of the radon-related radiation dose to humans is from the radon decay products polonium-218 and polonium-214 [13]. Decay products are primarily deposited on the surface of the respiratory tract, decaying in the lung due to their relatively short half-lives (<1 s-3 min) before being cleared by absorption into blood or particle transport to the gastrointestinal tract [12, 13]. Inhaled radon and decay products are predicted to deliver radiation doses to various tissues by virtue of irradiation by alpha particles emitted from radon decay products [13]. Alpha particles are particularly harmful, classified as having a high linear energy transfer (LET), reacting more readily with DNA and generating oxidative stress via radiolysis [17]. As radon gas is fat soluble, female breast tissue and red bone marrow receive high doses relative to other tissues [15]. The estimated annual radiation dose to the breast from inhalation of radon gas and decay products (i.e., polonium-218, lead-214, and bismuth-214) assuming a radon gas concentration of 200 Bq/m^3^ is 0.42 mSv and 0.02–0.15 mSv (depending on blood clearance rates), respectively, as compared to 1.2 mSv and 35.8–159 mSv for the lung [15]. Although these levels are low, the National Academy of Sciences’ Committee on Health Risks of Exposure to Radon (BEIR VI) report notes the possibility of radon-related DNA damage occurring at any level of radon exposure as a single alpha particle can cause substantial genetic damage to a cell [13].

Molecular and cellular studies have demonstrated that ionizing radiation emitted from the radioactive decay of radon and its decay products, primarily alpha particles, can cause cytogenetic damage, chromosome aberrations, and gene mutations [18]. Animal models suggest a potential link between radon and mammary tumors [19]. At the cellular level, alpha particles in the presence of estradiol were associated with increased cell proliferation and altered morphology in MCF-10F human breast cancer cells [20]. Moderate levels of radon (100 to 1200 μGy) have been associated with increased proliferation of MCF-7 human breast cancer cells [21, 22].

Although there is biological plausibility that radon exposure could influence breast carcinogenesis, few epidemiologic studies have been conducted. Increased breast cancer incidence was observed among former female employees of a Missouri school with elevated radon levels [23]. Ecologic studies showed no association between county-level radon levels and breast cancer incidence in the U.S. [24, 25]. A prospective analysis showed no association between radon exposure and breast cancer-related mortality [26]. Female breast cancer incidence was higher among residents of high-temperature geothermal areas characterized by radon-containing water in Iceland compared to residents of non-geothermal areas [27]. However, to date, no prospective epidemiologic study of breast cancer incidence has been conducted. The objective of this study was to examine the association between environmental radon exposure and breast cancer incidence in a prospective cohort of non-occupationally exposed U.S. women.

## Methods

### Study population

The Nurses’ Health Study II (NHSII) is an ongoing prospective cohort study of 116,429 U.S. female registered nurses aged 25–42 years at baseline in 1989. Although participants originally resided in California, Connecticut, Indiana, Iowa, Kentucky, Massachusetts, Michigan, Missouri, New York, North Carolina, Ohio, Pennsylvania, South Carolina, and Texas, as of the mid-1990’s, participants currently reside in all 50 states and the District of Columbia. Self-administered questionnaires are completed biennially acquiring information regarding incident disease, medical history, diet, lifestyle factors, and health behaviors. Response rates for each questionnaire cycle are ≥90%. We excluded women at baseline who were missing exposure information, with prior diagnoses of other cancers (except non-melanoma skin cancer), missing menopausal status, or who resided outside of the contiguous U.S. After exclusions, 112,639 women were included in the analysis. This study was approved by the Institutional Review Board of Brigham and Women’s Hospital; participants provided implied consent through returning questionnaires.

### Assessment of outcome

Invasive and in situ breast cancer cases were identified through self-report on biennial questionnaires. Deaths were reported by family members, the U.S. Postal Service, or ascertained from the National Death Index. A medical record review was conducted to confirm breast cancer cases and abstract information regarding tumor characteristics. As 99% of breast cancer cases were confirmed via medical record review, self-reported cases without medical record confirmation were also included in the analysis. Hormone receptor status was based on tissue microarrays (TMAs) constructed at the Dana-Farber/Harvard Cancer Center Tissue Microarray Core Facility. Three 0.6 mm diameter cores from tumor tissue samples were inserted into TMA blocks. Immunohistochemical staining for markers including estrogen receptor (ER) and progesterone receptor (PR) was performed on 5 μm paraffin sections cut from TMA blocks. Immunostained TMA sections were reviewed under a microscope and visually scored for ER and PR positivity as determined by any nuclear staining (≥1%) [28, 29]. Tumor cells were considered positive for human epidermal growth factor receptor 2 (HER2) protein overexpression when >10% of the cells showed moderate or strong membrane staining (2+ and 3+). If TMA information was unavailable, hormone receptor status was based on the medical record or pathology report. Our primary outcome of interest was incidence of invasive breast cancer and secondary outcomes of interest were invasive breast cancers defined by ER/PR/HER2 status.

### Exposure assessment

**Fig. 1:**
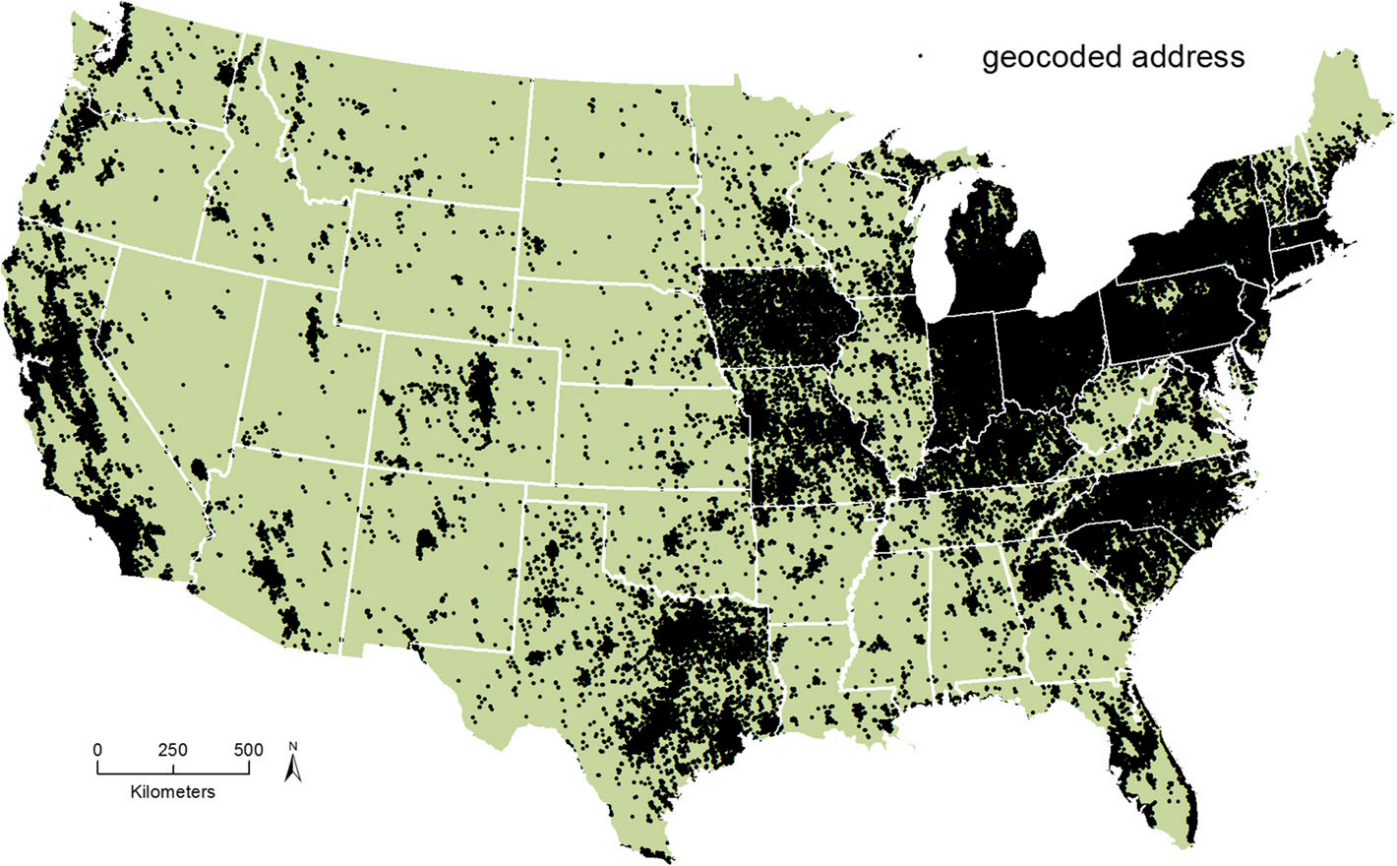
Nurses’ Health Study II geocoded residential addresses (1989–2011)

**Fig. 2:**
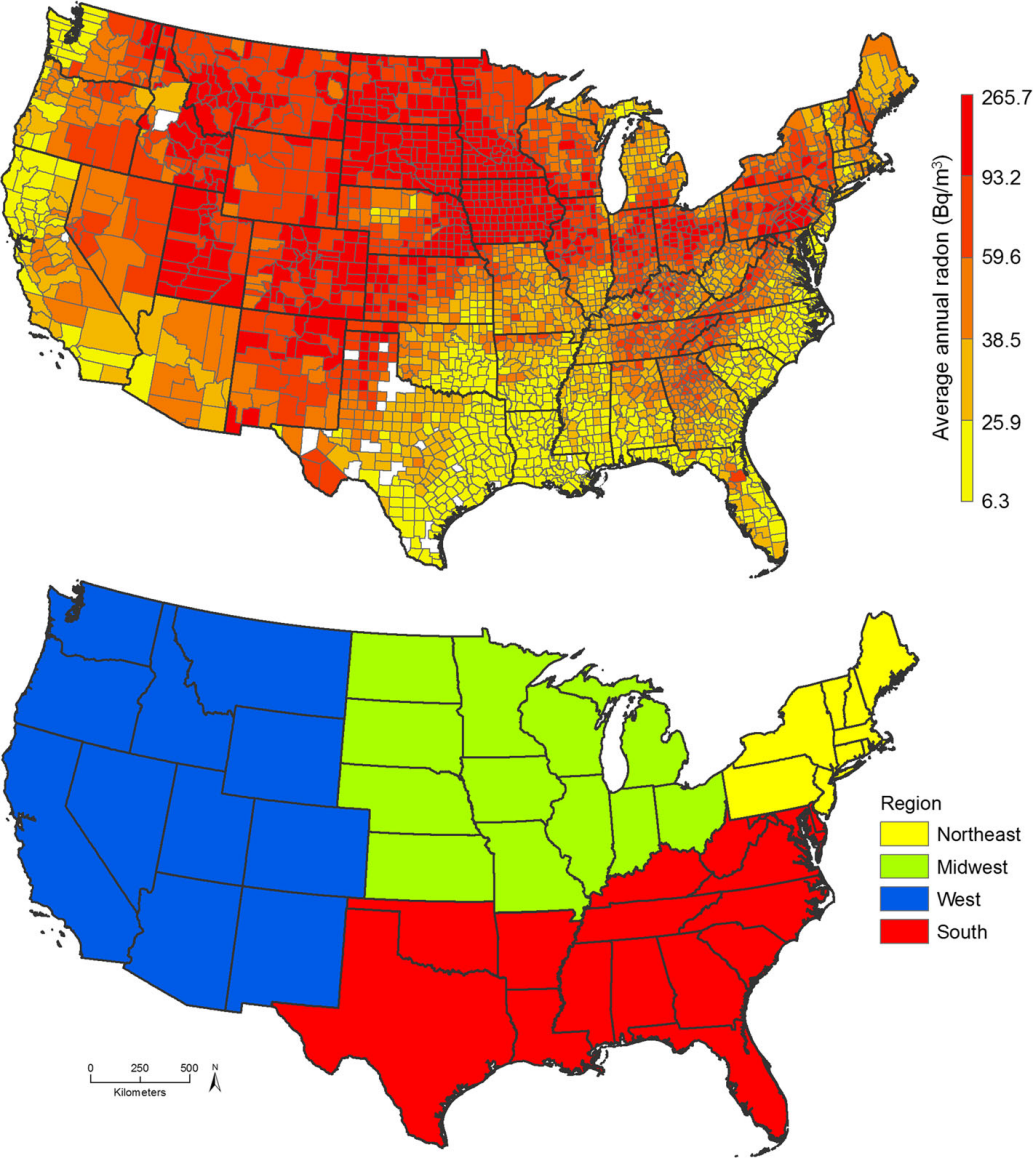
U.S. county-level average annual radon exposure (top) and U.S. Census Bureau regions (bottom)

Participant residential addresses, biennially updated beginning in 1989, were geocoded (Fig. 1) and spatially joined to the Lawrence Berkeley National Laboratory U.S. radon exposure model of county-level indoor radon concentrations (Fig. 2) in a geographic information system (GIS) using ArcMap 10.3.1 (Esri, Redlands, CA) [30]. The radon exposure model was calculated using Bayesian mixed-effects regression to predict average annual county-level radon concentrations derived from the short-term EPA/State Residential Radon Survey (SRRS) and long-term National Residential Radon Survey (NRRS) conducted during the mid- to late-1980s [31, 32]. The SRRS collected approximately 55,000 short-term winter screening measurements using charcoal canisters in 41 states. The NRRS collected approximately 60,000 annual average living area radon concentrations using alpha-track detectors and housing characteristics for approximately 5700 U.S. homes in 125 counties. Soil radium concentrations, geology (e.g., geologic provinces), housing characteristics (e.g., presence of basement), location of the screening measurement, meteorological data, and a conversion factor regarding the relationship between the SRRS and NRRS were included in the exposure model. The Lawrence Berkeley National Laboratory radon exposure model has been predictive of the adverse association between radon exposure and lung cancer in previous research [33]. Radon exposure for each participant was calculated as a time-varying cumulative average, where radon exposure from previous years was averaged and updated every two years over the course of follow-up [34]. In sensitivity analyses, we conducted analyses using only baseline (1989) radon exposure, defining exposure according to the EPA action level (≥148 Bq/m^3^), and estimating exposure using the University of Pittsburgh database of average radon levels (renormalized to the NRRS). The University of Pittsburgh database was created using approximately 272,000 residential radon measurements in 1217 counties from the University of Pittsburgh Radon Project (PITT), 40,000 EPA residential measurements in 39 states, and state-based studies in Florida, Idaho, Iowa, New Hampshire, New Jersey, New York, Ohio, South Carolina, and Utah [35].

### Additional covariates

The following information collected from biennial questionnaires (or every other questionnaire for diet and physical activity) were evaluated as potential confounders and/or effect modifiers, known to be established or suspected breast cancer risk factors: age, race, family history of breast cancer, personal history of biopsy-confirmed benign breast disease (BBD), age at menarche, parity and age at first birth, lactation, oral contraceptive (OC) use, menopausal status and hormone use (among postmenopausal women only), screening mammography, height, body mass index (BMI) at age 18, change in BMI since age 18, smoking status, diet scored by the Alternate Healthy Eating Index (AHEI), physical activity, alcohol consumption at age 15 and 18, adult alcohol consumption, and individual-level socioeconomic status (SES) (personal income, marital status, and living arrangements).

In addition, area-level SES (Census tract median home value and median income), region of residence, population density, county-level housing characteristics (percent of occupied housing units built after 1990; 1-unit detached/attached), and percentage of the population using well water from the U.S. Census Bureau, and fine (PM_2.5_) and coarse particulate matter (PM_2.5–10_) air pollution were determined using geocoded residential addresses. PM_2.5_ and PM_2.5–10_ were predicted using validated GIS-based spatiotemporal exposure models [36]. The missing indicator method was used to account for any missing covariates.

### Statistical analysis

Person-time accrued from June 1989 until the end of follow-up in May 2013, incidence of invasive breast cancer or other cancer (excluding non-melanoma skin cancer, but including in situ breast cancer), date of death, or loss to follow-up, whichever occurred first. Time-varying Cox regression models were used to calculate hazard ratios (HRs) and 95% confidence intervals (CIs) for the association between risk of incident invasive breast cancer and cumulative average radon exposure based on quintiles or continuous radon exposure per interquartile range (IQR) increase (37.3 Bq/m^3^). Tests for trend were calculated using the median value of each quintile of exposure. Cubic regression splines were used to test for deviations from linearity. All models were stratified by age and questionnaire period. Potential confounding was evaluated by adding each covariate (or group of covariates) to the model and noting its impact on the effect estimate for radon exposure (i.e., ≥10% change in the HR). Three models were assessed: a basic model minimally adjusted for age and race; a parsimonious model additionally adjusted for area- and individual-level SES and region of residence; and a fully adjusted model additionally controlling for established breast cancer risk factors (i.e., family history of breast cancer, screening mammography, BBD, reproductive and hormonal factors, height, adolescent and change in BMI, diet and lifestyle variables), PM_2.5_ air pollution, and population density. Results from shared frailty Cox models with a frailty term for county, which modifies the hazard multiplicatively and assigns each woman in a county (i.e., cluster) the same level of frailty, were compared to results from Cox models without a frailty term for county to determine the impact of within-county clustering [37].

We explored potential effect modification by race, menopausal status, age at first birth, parity, lactation, screening mammography, BMI, smoking status, Census tract median home value, Census tract median income, individual-level income, PM_2.5_, PM_2.5–10_, population density, urbanicity, and region of residence (Northeast, Midwest, West, and South; Fig. 2). States in each region are listed in Additional file 1: Table S1. These variables were considered as prior literature suggested differential associations with either radon or subgroups of the population with higher risk/susceptibility for breast cancer [38–46]. Effect modification was explored by conducting stratified analyses, where radon was categorized in tertiles rather than quintiles due to small numbers. Tests for interaction were performed by adding an interaction term to the model and using likelihood ratio tests to determine statistical significance. We performed sensitivity analyses among never-movers (i.e., participants who did not change residential addresses during follow-up). We calculated the *p*-value for heterogeneity comparing the association between radon exposure and invasive breast cancer risk by hormone receptor status (ER+; ER-; ER+/PR+; ER−/PR-; ER+/PR-; ER−/PR−/HER2- [triple-negative]) using partial likelihood ratio tests. All statistical analyses were conducted using SAS (SAS Institute, Cary, NC).

## Results

Table 1 presents characteristics of the 112,639 participants included in the analysis overall and by quintiles of cumulative average county-level radon over follow-up. The participants were on average 45.4 ± 8.3 years of age, mostly white, premenopausal, married, parous, and never-smokers. Women residing in areas with higher radon levels were more likely to be parous, have lower individual-level income, live in the Northeastern or Midwestern U.S., and live in areas with a lower Census tract median home value, income, and population density.

**Table 1:** Age-adjusted characteristics of *n* = 112,639 NHSII women over follow-up from 1989 to 2013 by cumulative average radon quintile

	Overall	Radon quintile 1 (<27.0 Bq/m^3^)	Radon quintile 2 (≥27.0–37.7 Bq/m^3^)	Radon quintile 3 (≥37.7–50.1 Bq/m^3^)	Radon quintile 4 (≥50.1–74.9 Bq/m^3^)	Radon quintile 5 (≥74.9 Bq/m^3^)
Person-years (*n*)	2,502,695	496,494	503,957	503,121	499,349	499,773
Cumulative average radon (Bq/m^3^) (mean ± SD)	54.7 ± 38.5	19.2 ± 3.9	32.2 ± 3.1	43.3 ± 3.5	61.5 ± 6.6	117.3 ± 38.5
Age (years) (mean ± SD)	45.4 ± 8.3	45.8 ± 8.2	45.3 ± 8.3	45.2 ± 8.3	45.4 ± 8.2	45.4 ± 8.3
White (%)	96	91	94	96	98	98
Family history of breast cancer (%)	11	11	11	11	11	10
Personal history of biopsy-confirmed benign breast disease (BBD) (%)	17	17	17	18	17	17
Menopausal status and hormone use (%)						
Premenopausal	67	67	67	68	67	67
Never users	5	4	5	5	5	5
Past users	11	11	11	11	11	11
Current users	8	9	8	7	7	8
Missing	9	10	10	9	9	9
Age at menarche (years) (mean ± SD)	12.4 ± 1.4	12.4 ± 1.5	12.4 ± 1.5	12.4 ± 1.4	12.4 ± 1.4	12.4 ± 1.4
Parity and age at first birth (%)						
Nulliparous	17	21	18	17	16	14
1–2 children <25	14	13	13	13	15	16
1–2 children ≥25–30	19	18	19	19	20	21
1–2 children ≥30	13	15	13	13	12	11
3+ children <25	11	8	10	12	12	14
3+ children ≥25–30	10	8	9	11	11	11
3+ children ≥30	2	2	3	3	2	2
Missing	13	15	14	13	12	11
Breastfeeding (%)						
Never	13	9	13	14	14	14
Ever	55	53	54	55	55	58
Missing	11	12	12	11	11	11
Oral contraceptive (OC) use (%)						
Never	12	10	12	13	13	11
Past	73	73	72	71	73	76
Current	7	7	7	6	6	6
Missing	9	10	10	9	8	7
Screening mammogram (%)						
No mammogram	22	22	22	22	22	22
Screening mammogram	52	51	51	53	52	53
Missing	26	28	27	26	26	25
Height (in) (mean ± SD)	64.9 ± 2.6	64.8 ± 2.7	64.8 ± 2.6	64.9 ± 2.6	64.8 ± 2.6	64.9 ± 2.6
Body mass index (BMI) (kg/m^2^) at age 18 (mean ± SD)	21.2 ± 3.2	21.0 ± 3.0	21.2 ± 3.1	21.3 ± 3.2	21.3 ± 3.2	21.4 ± 3.2
Current BMI (kg/m^2^) (mean ± SD)	25.5 ± 4.9	25.1 ± 4.8	25.4 ± 4.9	25.5 ± 4.9	25.6 ± 4.9	25.8 ± 5.0
Smoking status (%)						
Never	65	66	64	63	63	68
Past	25	25	26	26	25	22
Current	9	8	9	9	10	9
Alternate Healthy Eating Index (AHEI) (mean ± SD)	53.5 ± 9.8	55.3 ± 9.7	54.0 ± 9.7	53.4 ± 9.8	52.8 ± 9.7	52.0 ± 9.8
Cumulative average adult alcohol consumption (g/day) (mean ± SD)	3.2 ± 5.2	3.6 ± 5.9	3.3 ± 5.2	3.2 ± 5.1	3.0 ± 4.9	2.7 ± 4.8
Alcohol consumption at age 15 (g/day) (mean ± SD)	1.1 ± 3.6	1.1 ± 3.7	1.1 ± 3.6	1.2 ± 3.9	1.0 ± 3.5	0.9 ± 3.4
Alcohol consumption at age 18 (g/day) (mean ± SD)	5.1 ± 8.2	4.6 ± 7.8	5.0 ± 8.0	5.6 ± 8.5	5.4 ± 8.4	4.9 ± 8.0
Physical activity (MET hours/week) (mean ± SD)	19.8 ± 27.9	20.1 ± 28.4	19.8 ± 28.4	20.2 ± 28.7	19.7 ± 27.5	19.1 ± 26.4
Census tract median home value ($10,000) (mean ± SD)	16.3 ± 12.1	22.2 ± 16.2	18.2 ± 13.0	15.9 ± 11.7	13.3 ± 8.5	12.0 ± 5.3
Census tract median income ($) (mean ± SD)	63,669 ± 23,805	67,010 ± 26,620	66,984 ± 25,438	65,599 ± 24,124	60,849 ± 22,655	57,871 ± 17,755
Individual-level income >$100,000 (%)	21	25	23	22	19	18
Married (%)	56	52	55	56	57	60
Living alone (%)	7	8	7	7	7	6
Region of residence (%)						
Northeast	33	9	27	43	54	32
Midwest	32	1	30	41	30	59
West	15	51	15	5	3	2
South	19	38	29	11	12	7
Cumulative average PM_2.5_ (10 μg/m^3^) (mean ± SD)	1.5 ± 0.3	1.5 ± 0.4	1.5 ± 0.3	1.5 ± 0.3	1.6 ± 0.3	1.5 ± 0.3
Cumulative average PM_2.5–10_ (10 μg/m^3^) (mean ± SD)	1.1 ± 0.5	1.4 ± 0.6	1.1 ± 0.5	0.9 ± 0.4	0.9 ± 0.3	1.0 ± 0.4
Population density (per mi^2^) (mean ± SD)	3779 ± 10,885	3872 ± 5032	4432 ± 8188	6546 ± 20,144	2486 ± 8479	1533 ± 2252
Urbanicity (%)						
Urban	84	88	88	88	82	71
Urban Cluster	9	7	6	7	10	16
Small Town Rural	7	5	5	5	8	13
Percent of county occupied housing units: built after 1990 (mean ± SD)	23.1 ± 12.3	26.3 ± 13.4	23.4 ± 14.0	21.0 ± 11.7	20.6 ± 10.5	24.3 ± 10.3
Percent of county occupied housing units: single-unit detached/attached (mean ± SD)	71.4 ± 11.4	66.8 ± 8.5	70.6 ± 11.0	70.4 ± 16.8	73.3 ± 8.9	76.1 ± 6.8
Ever-moved (%)	79	83	81	79	75	76

During 2,502,695 person-years of follow-up from 1989 to 2013, 3966 invasive breast cancers occurred (*n* = 2373 ER+; *n* = 585 ER-; *n* = 2074 ER+/PR+; *n* = 513 ER−/PR-; *n* = 285 ER+/PR-; *n* = 293 ER−/PR−/HER2-). Increasing radon exposure was not associated with invasive breast cancer risk overall (adjusted HR comparing highest to lowest quintile = 1.06, 95% CI: 0.94, 1.21, *p* for trend = 0.30), ER+ cases, ER+/PR+ cases, or ER+/PR- cases in multivariable models (Table 2). The basic model adjusted for age and race showed a statistically significant association between higher radon exposure and risk of ER−/PR- breast cancer (*p* for trend = 0.01) and ER−/PR−/HER2- breast cancer (*p* for trend = 0.003) and a suggestive association for risk of ER- breast cancer (*p* for trend = 0.06). However, these associations were attenuated in fully adjusted models, primarily due to confounding by area-level socioeconomic status (see Additional file 1: Table S2 for detailed model building). Women in the highest quintile of exposure (≥74.9 Bq/m^3^) had a suggested elevated risk of ER- (adjusted HR = 1.34, 95% CI: 0.97, 1.86, *p* for trend = 0.15), ER−/PR- breast cancer (adjusted HR = 1.38, 95% CI: 0.97, 1.96, *p* for trend = 0.05), and ER−/PR−/HER2- breast cancer (adjusted HR = 1.52, 95% CI: 0.96, 2.41, *p* for trend = 0.02) compared to women in the lowest quintile (<27.0 Bq/m^3^) in fully adjusted models. Similar results were observed when examining radon exposure continuously (Table 2) and in analyses among premenopausal women only (Additional file 1: Table S3; the postmenopausal analysis is in Additional file 1: Table S4). There was no statistically significant heterogeneity in risk estimates by hormone receptor status (*p* > 0.05). Similar results were observed when using the University of Pittsburgh radon metric (results not shown), assessing baseline (1989) exposure (results not shown), defining exposure using the EPA action level of ≥148 Bq/m^3^ (Additional file 1: Table S5), and among never-movers (Additional file 1: Table S6). Results from shared frailty Cox models with a frailty term for county (that were able to converge) and Cox models without a frailty term for county were similar (results not shown).

**Table 2:** Associations between cumulative average radon and breast cancer risk in NHSII from 1989 to 2013 (n = 112,639)

Outcome^a^	Cases (*n*)	Person-years (*n*)	Basic^b^ HR (95% CI)	Fully adjusted^c^ HR (95% CI)
Invasive breast cancer				
Radon quintile 1	803	496,494	Referent	Referent
Radon quintile 2	810	503,957	1.03 (0.93, 1.13)	1.02 (0.92, 1.14)
Radon quintile 3	797	503,121	1.01 (0.92, 1.12)	1.02 (0.90, 1.14)
Radon quintile 4	745	499,349	0.94 (0.85, 1.04)	0.96 (0.85, 1.08)
Radon quintile 5	811	499,773	1.02 (0.93, 1.13)	1.06 (0.94, 1.21)
*p* for trend			0.96	0.30
Continuous radon (per IQR increase)^d^	3966	2,502,695	0.99 (0.96, 1.02)	1.01 (0.97, 1.04)
ER+				
Radon quintile 1	490	496,797	Referent	Referent
Radon quintile 2	471	504,288	0.98 (0.87, 1.12)	0.99 (0.87, 1.14)
Radon quintile 3	498	503,420	1.04 (0.92, 1.18)	1.07 (0.91, 1.24)
Radon quintile 4	439	499,621	0.91 (0.80, 1.03)	0.96 (0.82, 1.12)
Radon quintile 5	475	500,089	0.98 (0.86, 1.11)	1.05 (0.89, 1.23)
*p* for trend			0.53	0.66
Continuous radon (per IQR increase)^d^	2373	2,504,215	0.99 (0.95, 1.03)	1.01 (0.96, 1.05)
ER-				
Radon quintile 1	101	497,123	Referent	Referent
Radon quintile 2	112	504,612	1.11 (0.85, 1.46)	1.13 (0.84, 1.51)
Radon quintile 3	125	503,741	1.24 (0.96, 1.62)	1.27 (0.92, 1.74)
Radon quintile 4	114	499,939	1.14 (0.87, 1.49)	1.17 (0.84, 1.62)
Radon quintile 5	133	500,402	1.32 (1.02, 1.72)	1.34 (0.97, 1.86)
*p* for trend			0.06	0.15
Continuous radon (per IQR increase)^d^	585	2,505,817	1.05 (0.97, 1.13)	1.03 (0.95, 1.12)
ER+/PR+				
Radon quintile 1	417	496,859	Referent	Referent
Radon quintile 2	420	504,332	1.03 (0.90, 1.18)	1.06 (0.91, 1.23)
Radon quintile 3	441	503,469	1.09 (0.95, 1.24)	1.13 (0.96, 1.33)
Radon quintile 4	384	499,675	0.94 (0.81, 1.08)	1.01 (0.85, 1.19)
Radon quintile 5	412	500,148	1.00 (0.87, 1.15)	1.10 (0.92, 1.30)
*p* for trend			0.61	0.60
Continuous radon (per IQR increase)^d^	2074	2,504,483	0.99 (0.95, 1.04)	1.01 (0.97, 1.06)
ER−/PR-				
Radon quintile 1	89	497,137	Referent	Referent
Radon quintile 2	93	504,633	1.05 (0.79, 1.41)	1.07 (0.78, 1.46)
Radon quintile 3	104	503,763	1.18 (0.89, 1.57)	1.19 (0.85, 1.68)
Radon quintile 4	104	499,946	1.19 (0.89, 1.58)	1.20 (0.85, 1.69)
Radon quintile 5	123	500,409	1.41 (1.07, 1.85)	1.38 (0.97, 1.96)
*p* for trend			0.01	0.05
Continuous radon (per IQR increase)^d^	513	2,505,889	1.08 (0.99, 1.16)	1.05 (0.96, 1.15)
ER+/PR-				
Radon quintile 1	70	497,167	Referent	Referent
Radon quintile 2	47	504,679	0.69 (0.48, 1.00)	0.62 (0.41, 0.93)
Radon quintile 3	53	503,818	0.77 (0.54, 1.11)	0.66 (0.42, 1.02)
Radon quintile 4	53	499,984	0.76 (0.53, 1.09)	0.67 (0.43, 1.04)
Radon quintile 5	62	500,458	0.87 (0.61, 1.22)	0.75 (0.48, 1.18)
*p* for trend			0.88	1.00
Continuous radon (per IQR increase)^d^	285	2,506,105	0.97 (0.87, 1.09)	0.98 (0.86, 1.11)
ER−/PR−/HER2-				
Radon quintile 1	49	497,173	Referent	Referent
Radon quintile 2	53	504,676	1.09 (0.74, 1.60)	1.09 (0.71, 1.67)
Radon quintile 3	52	503,820	1.07 (0.72, 1.58)	1.06 (0.67, 1.68)
Radon quintile 4	61	499,980	1.25 (0.86, 1.83)	1.24 (0.78, 1.97)
Radon quintile 5	78	500,452	1.61 (1.12, 2.31)	1.52 (0.96, 2.41)
*p* for trend			0.003	0.02
Continuous radon (per IQR increase)^d^	293	2,506,101	1.12 (1.01, 1.24)	1.09 (0.97, 1.23)

^a^ Radon quintile 1: <27.0 Bq/m^3^; quintile 2: ≥27.0–37.7 Bq/m^3^; quintile 3: ≥37.7–50.1 Bq/m^3^; quintile 4: ≥50.1–74.9 Bq/m^3^; quintile 5: ≥74.9 Bq/m^3^

^b^ Adjusted for age, race

^c^ Additionally adjusted for Census tract median home value, Census tract median income, marital status, living arrangements, individual-level income, region of residence, family history of breast cancer, screening mammography, personal history of biopsy-confirmed BBD, age at menarche, parity, age at first birth, lactation, menopausal status and hormone use (among postmenopausal women only), height, BMI at age 18, change in BMI since age 18, smoking status, physical activity, adult alcohol consumption, PM_2.5_, population density

^d^ An IQR increase in cumulative average radon is 37.3 Bq/m^3^

There was a statistically significant interaction between radon exposure and region of residence (*p* = 0.0002), where among those residing in the Western U.S., women in the highest tertile of radon exposure (≥57.4 Bq/m^3^) had a 47% increased risk (95% CI: 1.10, 1.97) of invasive breast cancer compared to women in the lowest tertile (<33.3 Bq/m^3^) (Table 3). In comparison, radon exposure was not associated with increased risk of invasive breast cancer in the Northeast, Midwest, or South. There were no statistically significant differences in the association between radon exposure and invasive breast cancer risk by race, menopausal status, age at first birth, parity, lactation, screening mammography, BMI, smoking status, Census tract median home value, Census tract median income, individual-level income, PM_2.5_, PM_2.5–10_, urbanicity, well water use, and population density (results not shown).

**Table 3:** Cumulative average radon and breast cancer risk stratified by region of residence in NHSII

	Invasive breast cancer			ER+/PR+			ER−/PR-		
Stratification variable^a^	Cases/p-years	HR (95% CI)^b^	*p* int.	Cases/p-years	HR (95% CI)^b^	*p* int.	Cases/p-years	HR (95% CI)^b^	*p* int.
Region of residence			0.0002			0.13			0.45
Northeast									
Radon tertile 1	198/110,392	Referent		94/110,506	Referent		22/110,559	Referent	
Radon tertile 2	665/376,841	1.05 (0.90, 1.23)		356/377,134	1.19 (0.95, 1.50)		72/377,377	0.96 (0.60, 1.56)	
Radon tertile 3	494/341,830	0.83 (0.70, 0.99)		262/342,049	0.96 (0.76, 1.23)		69/342,242	0.91 (0.56, 1.49)	
Midwest									
Radon tertile 1	158/105,523	Referent		87/105,584	Referent		14/105,661	Referent	
Radon tertile 2	441/311,230	0.96 (0.80, 1.15)		241/311,433	0.95 (0.74, 1.21)		73/311,597	1.77 (1.00, 3.14)	
Radon tertile 3	616/391,334	1.09 (0.91, 1.29)		301/391,619	0.96 (0.76, 1.22)		96/391,793	1.86 (1.06, 3.26)	
West									
Radon tertile 1	504/308,674	Referent		279/308,883	Referent		62/309,064	Referent	
Radon tertile 2	94/48,620	1.19 (0.95, 1.49)		59/48,653	1.41 (1.06, 1.89)		8/48,713	0.72 (0.34, 1.53)	
Radon tertile 3	53/20,194	1.47 (1.10, 1.97)		23/20,223	1.22 (0.80, 1.88)		7/20,239	1.52 (0.69, 3.37)	
South									
Radon tertile 1	464/300,872	Referent		224/301,096	Referent		50/301,245	Referent	
Radon tertile 2	158/102,885	0.95 (0.79, 1.14)		76/102,968	0.95 (0.73, 1.23)		23/103,014	1.27 (0.77, 2.10)	
Radon tertile 3	110/77,355	0.91 (0.74, 1.13)		64/77,389	1.11 (0.84, 1.47)		17/77,433	1.26 (0.72, 2.19)	

Abbreviations: *p int. p*-value for interaction, *p-years* person-years

^a^ Radon tertile 1: <33.3 Bq/m^3^; tertile 2: ≥33.3–57.4 Bq/m^3^; tertile 3: ≥57.4 Bq/m^3^

^b^ Models are not adjusted for the stratifying variable. Models are adjusted for age, race, Census tract median home value, Census tract median income, marital status, living arrangements, individual-level income, family history of breast cancer, screening mammography, personal history of biopsy-confirmed BBD, age at menarche, parity, age at first birth, lactation, menopausal status and hormone use (among postmenopausal women only), height, BMI at age 18, change in BMI since age 18, smoking status, physical activity, adult alcohol consumption, PM_2.5_, population density

## Discussion

In this prospective analysis of U.S. female nurses, we observed suggestive associations between higher levels of exposure to county-level radon and risk of ER-, ER−/PR-, and ER−/PR−/HER2- invasive breast cancer after adjustment for variables including established breast cancer risk factors and socioeconomic factors. Radon was not associated with invasive breast cancer risk overall, ER+, ER+/PR+, or ER+/PR- breast cancer. We observed a statistically significant interaction between radon exposure and region of residence, where higher radon exposure was associated with an increased risk of invasive breast cancer mainly among women residing in the Western U.S. To the best of our knowledge, this is the first prospective analysis of environmental radon exposure and incident invasive breast cancer risk.

We observed a suggestive positive association between higher levels of radon exposure and risk of ER-, ER−/PR-, and ER−/PR−/HER2- invasive breast cancer. One possible explanation for this finding is that a potentially higher proportion of hormone receptor-positive vs. hormone receptor-negative tumors could be attributed to hormonal and reproductive risk factors [47]. Risk factor associations have been observed to differ by hormone receptor-positive vs. hormone receptor-negative breast cancer subtypes [48, 49]. Furthermore, ER−/PR- breast cancers are more common among women with a *BRCA1* mutation, which is involved in DNA repair pathways [50]. Thus, radon may be similarly acting on ER- carcinogenesis via DNA damage mechanisms. Further, ionizing radiation gives rise to significantly more ER- vs. ER+ tumors [51, 52]. Women with previously irradiated breast cancers (exposed to therapeutic radiation for Hodgkin lymphoma and other pediatric solid tumors) were more likely to have ER−/PR−/HER2- breast cancer compared to age-matched non-previously irradiated breast cancer controls [53]. However, in our study, there was no significant evidence of heterogeneity in risk estimates by hormone receptor status. The potential association between radon exposure and different breast cancer subtypes should be further explored.

There was a statistically significant interaction between radon exposure and region of residence, where higher radon exposure was significantly associated with an elevated risk of invasive breast cancer in the Western U.S. This interaction was not explained by differences in screening mammography practices, population density, urbanicity, residential mobility, PM air pollution, or area-level percentage of the population using well water, housing type, and year the housing was built. Radon was not strongly correlated with PM_2.5_ or PM_2.5–10_ overall (Spearman correlation coefficients 0.14 and −0.31, respectively) or in the West (−0.11 and −0.08). Further, although urban and rural differences in radon levels have been observed in previous research, partially attributed to differences in housing characteristics (e.g., construction) and dwelling habits (e.g., urban residents live on higher floors), we did not observe evidence of effect modification by urbanicity [54–56]. The majority of NHSII participants reside in urban areas (93%). There were also no significant regional differences in population characteristics or evidence of differential exposure assessment by region. Both the NRRS and SRRS surveys, used in Lawrence Berkeley National Laboratory radon exposure modeling, were designed to be representative of the entire U.S./states across the U.S., including the Western U.S. [57]. Among NHSII participants, radon levels were highest in the Midwest and Northeast, followed by the South and West – consistent with geographic patterns of radon levels across the U.S. observed in previous research [58]. However, some women residing in certain parts of the Western U.S. may be exposed to relatively higher levels of radon compared to women living in other regions, as parts of the Western U.S. are near active faults characterized by anomalously high radon emissions [59, 60]. Regional differences in exposure due to time spent indoors, individual-level housing characteristics (e.g., presence of basements), and remediation practices should be examined in future studies.

This analysis includes several important limitations. The radon exposure metric was available at a county-level spatial resolution, which may not reflect individual-level exposure. Household radon levels may vary within a county due to differences in housing characteristics, geology, and remediation. As counties are large geographic units often inhabited by many participants, we conducted sensitivity analyses accounting for within-county clustering using shared frailty models (frailty term for county). HRs from Cox models with and without frailty terms were similar. Exposure measurement errors are also likely due to errors in the radon exposure model’s failure to account for time-activity patterns by not having information available on amount of time spent at home, and lack of information on exposure to non-residential sources of radon including the workplace [61]. However, this radon exposure model has been used in previous epidemiologic studies of lung cancer, demonstrating expected positive associations, and may be considered a reasonable proxy of residential radon exposure [33]. The radon exposure data used in this study were collected during the mid- to late-1980s. We assumed radon levels remained consistent over time and calculated exposure measurements using updated address information throughout follow-up. However, radon levels can show high yearly variability given meteorological, diurnal, and seasonal changes [61, 62]. Advances in heating, ventilation, and air conditioning may have also impacted radon levels, as air conditioning use is associated with higher household radon levels [63]. However, long-term median household radon levels measured in 98 homes in the U.S. from 1983 to 2000 exhibited minimal year-to-year variation and no significant long-term temporal trends [64]. We did not have information on other factors that may account for varying exposures within a county, e.g., individual-level housing characteristics (e.g., floor of residence, presence of a basement), well water use, and remediation. We also did not have information regarding traffic noise, which has been associated with ER- breast cancer in Denmark [65]. The observed suggestive associations may be due to chance as we did not adjust our alpha level to account for multiple comparisons.

Strengths of this study include a long follow-up period of over 20 years allowing for a large number of breast cancer cases to accrue. To the best of our knowledge, this is the first prospective study assessing radon exposure and breast cancer risk. We objectively assessed radon exposure using a metric created from short- and long-term radon monitoring surveys, predictive of cancer risk in previous epidemiologic studies examining cancers [33]. Using this county-level radon exposure measure, we have also observed suggestive positive associations with lung cancer risk in the similarly designed Nurses’ Health Study (NHS) cohort that includes older participants (adjusted HR = 1.11, 95% CI: 0.67, 1.42) [66]. We were able to examine the association between radon and subtypes of breast cancer based on hormone receptor status, which is important as risk factor associations differ by subtype. NHSII collects time-varying information on established and suspected breast cancer risk factors, thus allowing for the opportunity to evaluate potential confounding and effect modification by many factors. Updated address information beginning in 1989 provided an opportunity to reconstruct historical radon exposure, which allowed us to evaluate long-term radon exposure and breast cancer incidence over a period of time spanning more than two decades, taking into account information regarding residential mobility.

## Conclusions

We observed suggestive positive associations between environmental radon exposure and ER-, ER−/PR-, and ER−/PR−/HER2- invasive breast cancer in a large prospective study of U.S. women, but no association overall, with ER+, ER+/PR+, or ER+/PR- invasive breast cancer. Further research is needed to clarify the association between radon exposure and invasive breast cancer risk, with a focus on hormone receptor-negative tumors, and to determine potential biological mechanisms.

## Additional file

Additional file 1: Supplemental tables for states in the U.S. Census Bureau regions, model building, associations between radon and breast cancer risk among premenopausal women, associations between radon and breast cancer risk among postmenopausal women, using the EPA radon action level, and stratified analyses by residential mobility. (DOCX 69 kb)

## Abbreviations

AHEI: Alternate Healthy Eating Index
BBD: Benign breast disease
BMI: Body mass index
CI: Confidence interval
EPA: Environmental Protection Agency
ER: Estrogen receptor
GIS: Geographic information system
HER2: Human epidermal growth factor receptor 2
HR: Hazard ratio
IARC: International Agency for Research on Cancer
IQR: Interquartile range
LET: Linear energy transfer
NHSII: Nurses’ Health Study II
NRRS: National Residential Radon Survey
OC: Oral contraceptive
PITT: University of Pittsburgh
PM_2.5_: Particulate matter <2.5 μm in diameter
PM_2.5–10_: Particulate matter 2.5–10 μm in diameter
PR: Progesterone receptor
SES: Socioeconomic status
SRRS: State Residential Radon Survey
TMA: Tissue microarray
