# Supplementary material for: Environmental radon exposure and breast cancer risk in the Nurses’ Health Study II
Source: Environ Health. 2017 Sep 7;16:97. doi: 10.1186/s12940-017-0305-6 (PMC5590193; doi:10.1186/s12940-017-0305-6)
Supplement: Additional file 1: — Supplemental tables for states in the U.S. Census Bureau regions, model building, associations between radon and breast cancer risk among premenopausal women, associations between radon and breast cancer risk among postmenopausal women, using the EPA radon action level, and stratified analyses by residential mobility. (DOCX 69 kb) [file 12940_2017_305_MOESM1_ESM.docx]

**Additional file 1** Supplemental tables for states in the U.S. Census Bureau regions, model building, associations between radon and breast cancer risk among premenopausal women, associations between radon and breast cancer risk among postmenopausal women, using the EPA radon action level, and stratified analyses by residential mobility

**Table 1** States located in U.S. Census Bureau regions (Northeast, Midwest, West, and South)

| Northeast | Midwest | West | South |
| --- | --- | --- | --- |
| Connecticut | Illinois | Alaska | Alabama |
| Maine | Indiana | Arizona | Arkansas |
| Massachusetts | Iowa | California | Delaware |
| New Hampshire | Kansas | Colorado | District of Columbia |
| New Jersey | Michigan | Hawaii | Florida |
| New York | Minnesota | Idaho | Georgia |
| Pennsylvania | Missouri | Montana | Kentucky |
| Rhode Island | Nebraska | Nevada | Louisiana |
| Vermont | North Dakota | New Mexico | Maryland |
|  | Ohio | Oregon | Mississippi |
|  | South Dakota | Utah | North Carolina |
|  | Wisconsin | Washington | Oklahoma |
|  |  | Wyoming | South Carolina |
|  |  |  | Tennessee |
|  |  |  | Texas |
|  |  |  | Virginia |
|  |  |  | West Virginia |

Note: States in the contiguous U.S. were included in the analysis.

**Table 2** Modeling the association between cumulative average radon and breast cancer risk in NHSII

|  | Invasive breast cancer | | ER+/PR+ | | ER-/PR- | |
| --- | --- | --- | --- | --- | --- | --- |
| Model^a^ | Cases/  person-years | HR (95% CI)^b^ | Cases/  person-years | HR (95% CI)^b^ | Cases/  person-years | HR (95% CI)^b^ |
| Age, race |  |  |  |  |  |  |
| Radon quintile 1 | 803/496,494 | Referent | 417/496,859 | Referent | 89/497,137 | Referent |
| Radon quintile 2 | 810/503,957 | 1.03 (0.93, 1.13) | 420/504,332 | 1.03 (0.90, 1.18) | 93/504,633 | 1.05 (0.79, 1.41) |
| Radon quintile 3 | 797/503,121 | 1.01 (0.92, 1.12) | 441/503,469 | 1.09 (0.95, 1.24) | 104/503,763 | 1.18 (0.89, 1.57) |
| Radon quintile 4 | 745/499,349 | 0.94 (0.85, 1.04) | 384/499,675 | 0.94 (0.81, 1.08) | 104/499,946 | 1.19 (0.89, 1.58) |
| Radon quintile 5 | 811/499,773 | 1.02 (0.93, 1.13) | 412/500,148 | 1.00 (0.87, 1.15) | 123/500,409 | 1.41 (1.07, 1.85) |
| *p* for trend |  | 0.96 |  | 0.61 |  | 0.01 |
| Continuous radon (per IQR increase)^c^ | 3,966/2,502,695 | 0.99 (0.96, 1.02) | 2,074/2,504,483 | 0.99 (0.95, 1.04) | 513/2,505,889 | 1.08 (0.99, 1.16) |
| Census tract median home value |  |  |  |  |  |  |
| Radon quintile 1 | 803/496,494 | Referent | 417/496,859 | Referent | 89/497,137 | Referent |
| Radon quintile 2 | 810/503,957 | 1.04 (0.95, 1.15) | 420/504,332 | 1.06 (0.92, 1.22) | 93/504,633 | 1.05 (0.78, 1.40) |
| Radon quintile 3 | 797/503,121 | 1.04 (0.94, 1.15) | 441/503,469 | 1.14 (0.99, 1.31) | 104/503,763 | 1.17 (0.88, 1.56) |
| Radon quintile 4 | 745/499,349 | 0.98 (0.89, 1.09) | 384/499,675 | 1.00 (0.87, 1.15) | 104/499,946 | 1.17 (0.87, 1.57) |
| Radon quintile 5 | 811/499,773 | 1.07 (0.97, 1.19) | 412/500,148 | 1.08 (0.94, 1.25) | 123/500,409 | 1.38 (1.04, 1.84) |
| *p* for trend |  | 0.34 |  | 0.58 |  | 0.02 |
| Continuous radon (per IQR increase)^c^ | 3,966/2,502,695 | 1.01 (0.98, 1.04) | 2,074/2,504,483 | 1.01 (0.97, 1.06) | 513/2,505,889 | 1.07 (0.98, 1.16) |
| Census tract median income |  |  |  |  |  |  |
| Radon quintile 1 | 803/496,494 | Referent | 417/496,859 | Referent | 89/497,137 | Referent |
| Radon quintile 2 | 810/503,957 | 1.02 (0.92, 1.13) | 420/504,332 | 1.04 (0.91, 1.20) | 93/504,633 | 1.02 (0.76, 1.37) |
| Radon quintile 3 | 797/503,121 | 1.01 (0.91, 1.12) | 441/503,469 | 1.12 (0.97, 1.28) | 104/503,763 | 1.13 (0.84, 1.52) |
| Radon quintile 4 | 745/499,349 | 0.95 (0.86, 1.06) | 384/499,675 | 0.98 (0.85, 1.13) | 104/499,946 | 1.13 (0.84, 1.53) |
| Radon quintile 5 | 811/499,773 | 1.05 (0.94, 1.16) | 412/500,148 | 1.06 (0.92, 1.23) | 123/500,409 | 1.35 (1.01, 1.80) |
| *p* for trend |  | 0.50 |  | 0.69 |  | 0.02 |
| Continuous radon (per IQR increase)^c^ | 3,966/2,502,695 | 1.00 (0.97, 1.03) | 2,074/2,504,483 | 1.01 (0.97, 1.06) | 513/2,505,889 | 1.06 (0.98, 1.15) |
| Marital status |  |  |  |  |  |  |
| Radon quintile 1 | 803/496,494 | Referent | 417/496,859 | Referent | 89/497,137 | Referent |
| Radon quintile 2 | 810/503,957 | 1.02 (0.92, 1.12) | 420/504,332 | 1.04 (0.90, 1.19) | 93/504,633 | 1.02 (0.76, 1.36) |
| Radon quintile 3 | 797/503,121 | 1.01 (0.91, 1.11) | 441/503,469 | 1.10 (0.96, 1.27) | 104/503,763 | 1.12 (0.84, 1.50) |
| Radon quintile 4 | 745/499,349 | 0.95 (0.85, 1.05) | 384/499,675 | 0.97 (0.84, 1.12) | 104/499,946 | 1.12 (0.83, 1.51) |
| Radon quintile 5 | 811/499,773 | 1.03 (0.93, 1.14) | 412/500,148 | 1.03 (0.90, 1.20) | 123/500,409 | 1.32 (0.99, 1.76) |
| *p* for trend |  | 0.74 |  | 0.99 |  | 0.03 |
| Continuous radon (per IQR increase)^c^ | 3,966/2,502,695 | 1.00 (0.97, 1.03) | 2,074/2,504,483 | 1.00 (0.96, 1.05) | 513/2,505,889 | 1.06 (0.97, 1.15) |
| Living arrangements |  |  |  |  |  |  |
| Radon quintile 1 | 803/496,494 | Referent | 417/496,859 | Referent | 89/497,137 | Referent |
| Radon quintile 2 | 810/503,957 | 1.02 (0.92, 1.12) | 420/504,332 | 1.04 (0.90, 1.19) | 93/504,633 | 1.02 (0.76, 1.37) |
| Radon quintile 3 | 797/503,121 | 1.01 (0.91, 1.11) | 441/503,469 | 1.11 (0.96, 1.27) | 104/503,763 | 1.12 (0.84, 1.50) |
| Radon quintile 4 | 745/499,349 | 0.95 (0.85, 1.05) | 384/499,675 | 0.97 (0.84, 1.12) | 104/499,946 | 1.12 (0.83, 1.51) |
| Radon quintile 5 | 811/499,773 | 1.03 (0.93, 1.14) | 412/500,148 | 1.04 (0.90, 1.20) | 123/500,409 | 1.32 (0.99, 1.76) |
| *p* for trend |  | 0.71 |  | 0.94 |  | 0.03 |
| Continuous radon (per IQR increase)^c^ | 3,966/2,502,695 | 1.00 (0.97, 1.03) | 2,074/2,504,483 | 1.01 (0.96, 1.05) | 513/2,505,889 | 1.06 (0.97, 1.15) |
| Individual-level income |  |  |  |  |  |  |
| Radon quintile 1 | 803/496,494 | Referent | 417/496,859 | Referent | 89/497,137 | Referent |
| Radon quintile 2 | 810/503,957 | 1.02 (0.92, 1.12) | 420/504,332 | 1.04 (0.90, 1.19) | 93/504,633 | 1.01 (0.75, 1.36) |
| Radon quintile 3 | 797/503,121 | 1.01 (0.91, 1.11) | 441/503,469 | 1.10 (0.96, 1.27) | 104/503,763 | 1.11 (0.83, 1.49) |
| Radon quintile 4 | 745/499,349 | 0.95 (0.85, 1.05) | 384/499,675 | 0.97 (0.84, 1.13) | 104/499,946 | 1.12 (0.83, 1.50) |
| Radon quintile 5 | 811/499,773 | 1.03 (0.93, 1.14) | 412/500,148 | 1.04 (0.90, 1.20) | 123/500,409 | 1.30 (0.98, 1.74) |
| *p* for trend |  | 0.72 |  | 0.92 |  | 0.04 |
| Continuous radon (per IQR increase)^c^ | 3,966/2,502,695 | 1.00 (0.97, 1.03) | 2,074/2,504,483 | 1.01 (0.96, 1.05) | 513/2,505,889 | 1.05 (0.97, 1.14) |
| Region of residence |  |  |  |  |  |  |
| Radon quintile 1 | 803/496,494 | Referent | 417/496,859 | Referent | 89/497,137 | Referent |
| Radon quintile 2 | 810/503,957 | 1.01 (0.91, 1.13) | 420/504,332 | 1.05 (0.90, 1.22) | 93/504,633 | 1.07 (0.78, 1.47) |
| Radon quintile 3 | 797/503,121 | 1.00 (0.89, 1.12) | 441/503,469 | 1.11 (0.94, 1.31) | 104/503,763 | 1.19 (0.85, 1.68) |
| Radon quintile 4 | 745/499,349 | 0.94 (0.83, 1.06) | 384/499,675 | 0.97 (0.82, 1.15) | 104/499,946 | 1.22 (0.86, 1.72) |
| Radon quintile 5 | 811/499,773 | 1.03 (0.91, 1.16) | 412/500,148 | 1.05 (0.88, 1.25) | 123/500,409 | 1.39 (0.98, 1.96) |
| *p* for trend |  | 0.66 |  | 0.99 |  | 0.05 |
| Continuous radon (per IQR increase)^c^ | 3,966/2,502,695 | 1.00 (0.96, 1.03) | 2,074/2,504,483 | 1.00 (0.96, 1.05) | 513/2,505,889 | 1.05 (0.96, 1.15) |
| Family history of breast cancer |  |  |  |  |  |  |
| Radon quintile 1 | 803/496,494 | Referent | 417/496,859 | Referent | 89/497,137 | Referent |
| Radon quintile 2 | 810/503,957 | 1.01 (0.91, 1.13) | 420/504,332 | 1.05 (0.90, 1.22) | 93/504,633 | 1.07 (0.78, 1.48) |
| Radon quintile 3 | 797/503,121 | 1.00 (0.89, 1.13) | 441/503,469 | 1.12 (0.95, 1.31) | 104/503,763 | 1.20 (0.85, 1.69) |
| Radon quintile 4 | 745/499,349 | 0.94 (0.83, 1.06) | 384/499,675 | 0.98 (0.82, 1.16) | 104/499,946 | 1.22 (0.87, 1.73) |
| Radon quintile 5 | 811/499,773 | 1.04 (0.92, 1.17) | 412/500,148 | 1.06 (0.89, 1.26) | 123/500,409 | 1.40 (0.99, 1.98) |
| *p* for trend |  | 0.57 |  | 0.94 |  | 0.04 |
| Continuous radon (per IQR increase)^c^ | 3,966/2,502,695 | 1.00 (0.96, 1.03) | 2,074/2,504,483 | 1.00 (0.96, 1.05) | 513/2,505,889 | 1.06 (0.97, 1.15) |
| Personal history of biopsy-confirmed BBD |  |  |  |  |  |  |
| Radon quintile 1 | 803/496,494 | Referent | 417/496,859 | Referent | 89/497,137 | Referent |
| Radon quintile 2 | 810/503,957 | 1.01 (0.91, 1.12) | 420/504,332 | 1.04 (0.90, 1.21) | 93/504,633 | 1.07 (0.78, 1.47) |
| Radon quintile 3 | 797/503,121 | 1.00 (0.88, 1.12) | 441/503,469 | 1.11 (0.94, 1.30) | 104/503,763 | 1.20 (0.85, 1.68) |
| Radon quintile 4 | 745/499,349 | 0.94 (0.83, 1.06) | 384/499,675 | 0.98 (0.82, 1.15) | 104/499,946 | 1.22 (0.87, 1.73) |
| Radon quintile 5 | 811/499,773 | 1.03 (0.91, 1.17) | 412/500,148 | 1.06 (0.89, 1.25) | 123/500,409 | 1.40 (0.99, 1.98) |
| *p* for trend |  | 0.56 |  | 0.92 |  | 0.04 |
| Continuous radon (per IQR increase)^c^ | 3,966/2,502,695 | 1.00 (0.97, 1.03) | 2,074/2,504,483 | 1.00 (0.96, 1.05) | 513/2,505,889 | 1.06 (0.97, 1.16) |
| Reproductive factors: age at menarche, parity, age at first birth, lactation, menopausal status and hormone use (among postmenopausal women only) |  |  |  |  |  |  |
| Radon quintile 1 | 803/496,494 | Referent | 417/496,859 | Referent | 89/497,137 | Referent |
| Radon quintile 2 | 810/503,957 | 1.02 (0.91, 1.13) | 420/504,332 | 1.05 (0.91, 1.22) | 93/504,633 | 1.07 (0.78, 1.46) |
| Radon quintile 3 | 797/503,121 | 1.00 (0.89, 1.13) | 441/503,469 | 1.12 (0.95, 1.32) | 104/503,763 | 1.19 (0.85, 1.68) |
| Radon quintile 4 | 745/499,349 | 0.95 (0.84, 1.07) | 384/499,675 | 0.99 (0.84, 1.17) | 104/499,946 | 1.21 (0.86, 1.71) |
| Radon quintile 5 | 811/499,773 | 1.05 (0.93, 1.18) | 412/500,148 | 1.07 (0.90, 1.28) | 123/500,409 | 1.38 (0.98, 1.96) |
| *p* for trend |  | 0.44 |  | 0.77 |  | 0.05 |
| Continuous radon (per IQR increase)^c^ | 3,966/2,502,695 | 1.00 (0.97, 1.04) | 2,074/2,504,483 | 1.01 (0.96, 1.06) | 513/2,505,889 | 1.05 (0.96, 1.15) |
| Screening mammography |  |  |  |  |  |  |
| Radon quintile 1 | 803/496,494 | Referent | 417/496,859 | Referent | 89/497,137 | Referent |
| Radon quintile 2 | 810/503,957 | 1.02 (0.91, 1.13) | 420/504,332 | 1.05 (0.91, 1.22) | 93/504,633 | 1.07 (0.78, 1.47) |
| Radon quintile 3 | 797/503,121 | 1.00 (0.89, 1.13) | 441/503,469 | 1.12 (0.95, 1.32) | 104/503,763 | 1.19 (0.85, 1.68) |
| Radon quintile 4 | 745/499,349 | 0.95 (0.84, 1.07) | 384/499,675 | 0.99 (0.84, 1.18) | 104/499,946 | 1.21 (0.86, 1.71) |
| Radon quintile 5 | 811/499,773 | 1.05 (0.93, 1.18) | 412/500,148 | 1.08 (0.90, 1.28) | 123/500,409 | 1.39 (0.98, 1.96) |
| *p* for trend |  | 0.44 |  | 0.77 |  | 0.05 |
| Continuous radon (per IQR increase)^c^ | 3,966/2,502,695 | 1.00 (0.97, 1.04) | 2,074/2,504,483 | 1.01 (0.96, 1.06) | 513/2,505,889 | 1.05 (0.96, 1.15) |
| Height, BMI at age 18, change in BMI since age 18 |  |  |  |  |  |  |
| Radon quintile 1 | 803/496,494 | Referent | 417/496,859 | Referent | 89/497,137 | Referent |
| Radon quintile 2 | 810/503,957 | 1.02 (0.91, 1.13) | 420/504,332 | 1.06 (0.91, 1.23) | 93/504,633 | 1.07 (0.78, 1.46) |
| Radon quintile 3 | 797/503,121 | 1.01 (0.89, 1.13) | 441/503,469 | 1.12 (0.95, 1.32) | 104/503,763 | 1.19 (0.85, 1.67) |
| Radon quintile 4 | 745/499,349 | 0.95 (0.84, 1.07) | 384/499,675 | 1.00 (0.84, 1.18) | 104/499,946 | 1.21 (0.86, 1.71) |
| Radon quintile 5 | 811/499,773 | 1.05 (0.93, 1.19) | 412/500,148 | 1.08 (0.91, 1.29) | 123/500,409 | 1.39 (0.98, 1.96) |
| *p* for trend |  | 0.38 |  | 0.71 |  | 0.04 |
| Continuous radon (per IQR increase)^c^ | 3,966/2,502,695 | 1.00 (0.97, 1.04) | 2,074/2,504,483 | 1.01 (0.96, 1.06) | 513/2,505,889 | 1.05 (0.96, 1.15) |
| Lifestyle factors: smoking status, physical activity, adult alcohol consumption |  |  |  |  |  |  |
| Radon quintile 1 | 803/496,494 | Referent | 417/496,859 | Referent | 89/497,137 | Referent |
| Radon quintile 2 | 810/503,957 | 1.02 (0.92, 1.14) | 420/504,332 | 1.06 (0.91, 1.23) | 93/504,633 | 1.06 (0.77, 1.46) |
| Radon quintile 3 | 797/503,121 | 1.01 (0.89, 1.13) | 441/503,469 | 1.12 (0.95, 1.32) | 104/503,763 | 1.19 (0.85, 1.67) |
| Radon quintile 4 | 745/499,349 | 0.95 (0.85, 1.08) | 384/499,675 | 1.00 (0.84, 1.18) | 104/499,946 | 1.21 (0.85, 1.70) |
| Radon quintile 5 | 811/499,773 | 1.06 (0.94, 1.20) | 412/500,148 | 1.09 (0.92, 1.29) | 123/500,409 | 1.39 (0.98, 1.97) |
| *p* for trend |  | 0.31 |  | 0.64 |  | 0.04 |
| Continuous radon (per IQR increase)^c^ | 3,966/2,502,695 | 1.01 (0.97, 1.04) | 2,074/2,504,483 | 1.01 (0.97, 1.06) | 513/2,505,889 | 1.05 (0.96, 1.15) |
| PM_2.5_ air pollution |  |  |  |  |  |  |
| Radon quintile 1 | 803/496,494 | Referent | 417/496,859 | Referent | 89/497,137 | Referent |
| Radon quintile 2 | 810/503,957 | 1.02 (0.92, 1.14) | 420/504,332 | 1.05 (0.91, 1.22) | 93/504,633 | 1.06 (0.77, 1.46) |
| Radon quintile 3 | 797/503,121 | 1.01 (0.90, 1.14) | 441/503,469 | 1.13 (0.96, 1.33) | 104/503,763 | 1.19 (0.84, 1.67) |
| Radon quintile 4 | 745/499,349 | 0.96 (0.85, 1.09) | 384/499,675 | 1.01 (0.85, 1.19) | 104/499,946 | 1.20 (0.85, 1.70) |
| Radon quintile 5 | 811/499,773 | 1.07 (0.94, 1.21) | 412/500,148 | 1.10 (0.92, 1.30) | 123/500,409 | 1.38 (0.98, 1.96) |
| *p* for trend |  | 0.26 |  | 0.58 |  | 0.04 |
| Continuous radon (per IQR increase)^c^ | 3,966/2,502,695 | 1.01 (0.97, 1.04) | 2,074/2,504,483 | 1.01 (0.97, 1.06) | 513/2,505,889 | 1.05 (0.96, 1.15) |
| Population density |  |  |  |  |  |  |
| Radon quintile 1 | 803/496,494 | Referent | 417/496,859 | Referent | 89/497,137 | Referent |
| Radon quintile 2 | 810/503,957 | 1.02 (0.92, 1.14) | 420/504,332 | 1.06 (0.91, 1.23) | 93/504,633 | 1.07 (0.78, 1.46) |
| Radon quintile 3 | 797/503,121 | 1.02 (0.90, 1.14) | 441/503,469 | 1.13 (0.96, 1.33) | 104/503,763 | 1.19 (0.85, 1.68) |
| Radon quintile 4 | 745/499,349 | 0.96 (0.85, 1.08) | 384/499,675 | 1.01 (0.85, 1.19) | 104/499,946 | 1.20 (0.85, 1.69) |
| Radon quintile 5 | 811/499,773 | 1.06 (0.94, 1.21) | 412/500,148 | 1.10 (0.92, 1.30) | 123/500,409 | 1.38 (0.97, 1.96) |
| *p* for trend |  | 0.30 |  | 0.60 |  | 0.05 |
| Continuous radon (per IQR increase)^c^ | 3,966/2,502,695 | 1.01 (0.97, 1.04) | 2,074/2,504,483 | 1.01 (0.97, 1.06) | 513/2,505,889 | 1.05 (0.96, 1.15) |

^a^ Radon quintile 1: <27.0 Bq/m^3^; quintile 2: ≥27.0-37.7 Bq/m^3^; quintile 3: ≥37.7-50.1 Bq/m^3^; quintile 4: ≥50.1-74.9 Bq/m^3^; quintile 5: ≥74.9 Bq/m^3^.

^b^ Each model additionally adjusts for the variables in the previous models.

^c^ An IQR increase in cumulative average radon is 37.3 Bq/m^3^.

**Table 3** Associations between cumulative average radon and breast cancer risk in NHSII among premenopausal women

| Outcome^a^ | Cases/person-years | Basic^b^  HR (95% CI) | Fully adjusted^c^  HR (95% CI) |
| --- | --- | --- | --- |
| Invasive breast cancer |  |  |  |
| Radon quintile 1 | 448/328,764 | Referent | Referent |
| Radon quintile 2 | 469/342,842 | 1.03 (0.91, 1.18) | 1.00 (0.86, 1.15) |
| Radon quintile 3 | 472/347,464 | 1.02 (0.90, 1.17) | 0.97 (0.83, 1.14) |
| Radon quintile 4 | 424/338,931 | 0.93 (0.81, 1.06) | 0.89 (0.75, 1.05) |
| Radon quintile 5 | 443/337,910 | 0.98 (0.86, 1.12) | 0.98 (0.83, 1.16) |
| *p* for trend |  | 0.42 | 0.83 |
| Continuous radon (per IQR increase)^d^ | 2,256/1,695,911 | 0.97 (0.93, 1.01) | 0.98 (0.94, 1.03) |
| ER+ |  |  |  |
| Radon quintile 1 | 285/328,919 | Referent | Referent |
| Radon quintile 2 | 288/343,011 | 1.00 (0.85, 1.18) | 0.95 (0.79, 1.14) |
| Radon quintile 3 | 311/347,626 | 1.07 (0.91, 1.25) | 0.99 (0.81, 1.21) |
| Radon quintile 4 | 270/339,056 | 0.92 (0.78, 1.09) | 0.87 (0.71, 1.08) |
| Radon quintile 5 | 284/338,043 | 0.98 (0.83, 1.16) | 0.97 (0.78, 1.20) |
| *p* for trend |  | 0.56 | 0.88 |
| Continuous radon (per IQR increase)^d^ | 1,438/1,696,655 | 0.99 (0.94, 1.04) | 1.00 (0.95, 1.06) |
| ER- |  |  |  |
| Radon quintile 1 | 61/329,096 | Referent | Referent |
| Radon quintile 2 | 81/343,192 | 1.29 (0.92, 1.80) | 1.48 (1.03, 2.13) |
| Radon quintile 3 | 85/347,816 | 1.34 (0.96, 1.87) | 1.63 (1.09, 2.43) |
| Radon quintile 4 | 69/339,251 | 1.12 (0.79, 1.59) | 1.36 (0.90, 2.08) |
| Radon quintile 5 | 82/338,226 | 1.34 (0.96, 1.87) | 1.65 (1.09, 2.51) |
| *p* for trend |  | 0.29 | 0.19 |
| Continuous radon (per IQR increase)^d^ | 378/1,697,580 | 1.02 (0.92, 1.12) | 1.03 (0.92, 1.14) |
| ER+/PR+ |  |  |  |
| Radon quintile 1 | 247/328,950 | Referent | Referent |
| Radon quintile 2 | 264/343,030 | 1.06 (0.89, 1.27) | 1.02 (0.84, 1.24) |
| Radon quintile 3 | 279/347,651 | 1.11 (0.93, 1.32) | 1.06 (0.85, 1.31) |
| Radon quintile 4 | 243/339,086 | 0.96 (0.81, 1.15) | 0.95 (0.76, 1.18) |
| Radon quintile 5 | 253/338,072 | 1.01 (0.85, 1.21) | 1.03 (0.83, 1.29) |
| *p* for trend |  | 0.71 | 0.94 |
| Continuous radon (per IQR increase)^d^ | 1,286/1,696,789 | 1.00 (0.94, 1.05) | 1.01 (0.95, 1.07) |
| ER-/PR- |  |  |  |
| Radon quintile 1 | 52/329,107 | Referent | Referent |
| Radon quintile 2 | 66/343,208 | 1.23 (0.86, 1.78) | 1.42 (0.95, 2.12) |
| Radon quintile 3 | 68/347,833 | 1.26 (0.88, 1.82) | 1.50 (0.97, 2.33) |
| Radon quintile 4 | 63/339,254 | 1.21 (0.84, 1.76) | 1.42 (0.90, 2.22) |
| Radon quintile 5 | 75/338,231 | 1.46 (1.02, 2.09) | 1.70 (1.08, 2.67) |
| *p* for trend |  | 0.06 | 0.09 |
| Continuous radon (per IQR increase)^d^ | 324/1,697,632 | 1.05 (0.95, 1.16) | 1.04 (0.93, 1.17) |
| ER+/PR- |  |  |  |
| Radon quintile 1 | 38/329,126 | Referent | Referent |
| Radon quintile 2 | 21/343,246 | 0.54 (0.32, 0.92) | 0.42 (0.23, 0.77) |
| Radon quintile 3 | 29/347,875 | 0.72 (0.44, 1.17) | 0.48 (0.26, 0.90) |
| Radon quintile 4 | 25/339,277 | 0.59 (0.35, 0.98) | 0.40 (0.21, 0.76) |
| Radon quintile 5 | 30/338,263 | 0.73 (0.45, 1.18) | 0.51 (0.27, 0.97) |
| *p* for trend |  | 0.53 | 0.48 |
| Continuous radon (per IQR increase)^d^ | 143/1,697,786 | 0.96 (0.82, 1.14) | 0.96 (0.79, 1.15) |
| ER-/PR-/HER2- |  |  |  |
| Radon quintile 1 | 29/329,127 | Referent | Referent |
| Radon quintile 2 | 36/343,236 | 1.19 (0.73, 1.94) | 1.45 (0.85, 2.48) |
| Radon quintile 3 | 32/347,871 | 1.05 (0.63, 1.74) | 1.36 (0.74, 2.48) |
| Radon quintile 4 | 34/339,278 | 1.15 (0.70, 1.89) | 1.51 (0.83, 2.77) |
| Radon quintile 5 | 44/338,258 | 1.50 (0.93, 2.41) | 1.92 (1.05, 3.50) |
| *p* for trend |  | 0.08 | 0.05 |
| Continuous radon (per IQR increase)^d^ | 175/1,697,769 | 1.05 (0.92, 1.21) | 1.07 (0.92, 1.25) |

^a^ Radon quintile 1: <27.0 Bq/m^3^; quintile 2: ≥27.0-37.7 Bq/m^3^; quintile 3: ≥37.7-50.1 Bq/m^3^; quintile 4: ≥50.1-74.9 Bq/m^3^; quintile 5: ≥74.9 Bq/m^3^.

^b^ Adjusted for age, race.

^c^ Additionally adjusted for Census tract median home value, Census tract median income, marital status, living arrangements, individual-level income, region of residence, family history of breast cancer, screening mammography, personal history of biopsy-confirmed BBD, age at menarche, parity, age at first birth, lactation, height, BMI at age 18, change in BMI since age 18, smoking status, physical activity, adult alcohol consumption, PM_2.5_ air pollution, population density.

^d^ An IQR increase in cumulative average radon is 37.3 Bq/m^3^.

**Table 4** Associations between cumulative average radon and breast cancer risk in NHSII among postmenopausal women

| Outcome^a^ | Cases/person-years | Basic^b^  HR (95% CI) | Fully adjusted^c^  HR (95% CI) |
| --- | --- | --- | --- |
| Invasive breast cancer |  |  |  |
| Radon quintile 1 | 340/162,780 | Referent | Referent |
| Radon quintile 2 | 318/156,431 | 0.98 (0.84, 1.15) | 1.02 (0.87, 1.21) |
| Radon quintile 3 | 312/150,249 | 1.00 (0.85, 1.16) | 1.07 (0.89, 1.28) |
| Radon quintile 4 | 302/155,655 | 0.93 (0.80, 1.09) | 1.02 (0.84, 1.23) |
| Radon quintile 5 | 354/158,131 | 1.07 (0.92, 1.24) | 1.16 (0.96, 1.41) |
| *p* for trend |  | 0.37 | 0.08 |
| Continuous radon (per IQR increase)^d^ | 1,626/783,245 | 1.02 (0.97, 1.06) | 1.04 (0.98, 1.09) |
| ER+ |  |  |  |
| Radon quintile 1 | 195/162,920 | Referent | Referent |
| Radon quintile 2 | 168/156,582 | 0.93 (0.75, 1.14) | 1.02 (0.81, 1.27) |
| Radon quintile 3 | 184/150,376 | 1.03 (0.84, 1.27) | 1.21 (0.95, 1.54) |
| Radon quintile 4 | 160/155,787 | 0.87 (0.71, 1.08) | 1.06 (0.82, 1.36) |
| Radon quintile 5 | 186/158,299 | 0.99 (0.80, 1.21) | 1.18 (0.92, 1.53) |
| *p* for trend |  | 0.88 | 0.28 |
| Continuous radon (per IQR increase)^d^ | 893/783,964 | 0.99 (0.93, 1.05) | 1.02 (0.95, 1.10) |
| ER- |  |  |  |
| Radon quintile 1 | 35/163,063 | Referent | Referent |
| Radon quintile 2 | 35/156,705 | 1.02 (0.64, 1.63) | 0.88 (0.53, 1.47) |
| Radon quintile 3 | 38/150,508 | 1.16 (0.73, 1.84) | 0.92 (0.53, 1.60) |
| Radon quintile 4 | 42/155,903 | 1.18 (0.75, 1.86) | 0.95 (0.55, 1.64) |
| Radon quintile 5 | 43/158,434 | 1.17 (0.74, 1.83) | 0.89 (0.51, 1.57) |
| *p* for trend |  | 0.47 | 0.82 |
| Continuous radon (per IQR increase)^d^ | 193/784,614 | 1.03 (0.91, 1.18) | 0.97 (0.83, 1.13) |
| ER+/PR+ |  |  |  |
| Radon quintile 1 | 162/162,951 | Referent | Referent |
| Radon quintile 2 | 142/156,606 | 0.94 (0.75, 1.18) | 1.04 (0.81, 1.33) |
| Radon quintile 3 | 160/150,399 | 1.08 (0.87, 1.34) | 1.27 (0.97, 1.64) |
| Radon quintile 4 | 132/155,812 | 0.86 (0.68, 1.08) | 1.05 (0.79, 1.38) |
| Radon quintile 5 | 155/158,331 | 0.99 (0.80, 1.24) | 1.20 (0.91, 1.58) |
| *p* for trend |  | 0.85 | 0.36 |
| Continuous radon (per IQR increase)^d^ | 751/784,100 | 1.00 (0.93, 1.07) | 1.03 (0.96, 1.12) |
| ER-/PR- |  |  |  |
| Radon quintile 1 | 33/163,065 | Referent | Referent |
| Radon quintile 2 | 31/156,711 | 0.96 (0.58, 1.57) | 0.87 (0.51, 1.49) |
| Radon quintile 3 | 33/150,515 | 1.07 (0.66, 1.74) | 0.94 (0.53, 1.68) |
| Radon quintile 4 | 38/155,906 | 1.13 (0.71, 1.82) | 1.00 (0.56, 1.76) |
| Radon quintile 5 | 41/158,435 | 1.19 (0.75, 1.88) | 0.99 (0.55, 1.78) |
| *p* for trend |  | 0.35 | 0.79 |
| Continuous radon (per IQR increase)^d^ | 176/784,631 | 1.06 (0.92, 1.21) | 1.02 (0.87, 1.19) |
| ER+/PR- |  |  |  |
| Radon quintile 1 | 30/163,071 | Referent | Referent |
| Radon quintile 2 | 25/156,720 | 0.91 (0.53, 1.55) | 0.92 (0.52, 1.65) |
| Radon quintile 3 | 23/150,523 | 0.86 (0.50, 1.48) | 0.96 (0.50, 1.84) |
| Radon quintile 4 | 28/155,916 | 1.02 (0.61, 1.72) | 1.21 (0.64, 2.30) |
| Radon quintile 5 | 31/158,445 | 1.04 (0.63, 1.73) | 1.17 (0.61, 2.24) |
| *p* for trend |  | 0.67 | 0.43 |
| Continuous radon (per IQR increase)^d^ | 137/784,674 | 0.95 (0.80, 1.12) | 0.96 (0.79, 1.16) |
| ER-/PR-/HER2- |  |  |  |
| Radon quintile 1 | 19/163,078 | Referent | Referent |
| Radon quintile 2 | 20/156,725 | 1.08 (0.57, 2.02) | 0.89 (0.45, 1.77) |
| Radon quintile 3 | 19/150,529 | 1.06 (0.56, 2.02) | 0.75 (0.36, 1.59) |
| Radon quintile 4 | 25/155,915 | 1.26 (0.69, 2.30) | 0.85 (0.41, 1.76) |
| Radon quintile 5 | 28/158,450 | 1.40 (0.78, 2.52) | 0.85 (0.41, 1.80) |
| *p* for trend |  | 0.20 | 0.92 |
| Continuous radon (per IQR increase)^d^ | 111/784,697 | 1.12 (0.95, 1.32) | 1.02 (0.84, 1.23) |

^a^ Radon quintile 1: <27.0 Bq/m^3^; quintile 2: ≥27.0-37.7 Bq/m^3^; quintile 3: ≥37.7-50.1 Bq/m^3^; quintile 4: ≥50.1-74.9 Bq/m^3^; quintile 5: ≥74.9 Bq/m^3^.

^b^ Adjusted for age, race.

^c^ Additionally adjusted for Census tract median home value, Census tract median income, marital status, living arrangements, individual-level income, region of residence, family history of breast cancer, screening mammography, personal history of biopsy-confirmed BBD, age at menarche, parity, age at first birth, lactation, hormone use (among postmenopausal women only), height, BMI at age 18, change in BMI since age 18, smoking status, physical activity, adult alcohol consumption, PM_2.5_ air pollution, population density.

^d^ An IQR increase in cumulative average radon is 37.3 Bq/m^3^.

**Table 5** Cumulative average radon exposure above the EPA action level (≥148 Bq/m^3^) and breast cancer risk

| Outcome | Cases/person-years | Basic^a^  HR (95% CI) | p | Fully adjusted^b^  HR (95% CI) | p |
| --- | --- | --- | --- | --- | --- |
| Invasive breast cancer |  |  |  |  |  |
| Radon exposure <148 Bq/m^3^ | 3,820/2,406,011 | Referent |  | Referent |  |
| Radon exposure ≥148 Bq/m^3^ | 146/96,684 | 0.94 (0.80, 1.11) | 0.47 | 0.95 (0.80, 1.12) | 0.53 |
| ER+ |  |  |  |  |  |
| Radon exposure <148 Bq/m^3^ | 2,276/2,407,477 | Referent |  | Referent |  |
| Radon exposure ≥148 Bq/m^3^ | 97/96,738 | 1.04 (0.85, 1.28) | 0.70 | 1.06 (0.86, 1.30) | 0.70 |
| ER- |  |  |  |  |  |
| Radon exposure <148 Bq/m^3^ | 568/2,409,004 | Referent |  | Referent |  |
| Radon exposure ≥148 Bq/m^3^ | 17/96,813 | 0.74 (0.46, 1.21) | 0.23 | 0.71 (0.43, 1.15) | 0.16 |
| ER+/PR+ |  |  |  |  |  |
| Radon exposure <148 Bq/m^3^ | 1,986/2,407,734 | Referent |  | Referent |  |
| Radon exposure ≥148 Bq/m^3^ | 88/96,749 | 1.08 (0.87, 1.34) | 0.47 | 1.10 (0.88, 1.36) | 0.40 |
| ER-/PR- |  |  |  |  |  |
| Radon exposure <148 Bq/m^3^ | 497/2,409,075 | Referent |  | Referent |  |
| Radon exposure ≥148 Bq/m^3^ | 16/96,814 | 0.81 (0.49, 1.34) | 0.42 | 0.75 (0.45, 1.25) | 0.27 |
| ER+/PR- |  |  |  |  |  |
| Radon exposure <148 Bq/m^3^ | 276/2,409,286 | Referent |  | Referent |  |
| Radon exposure ≥148 Bq/m^3^ | 9/96,819 | 0.80 (0.41, 1.55) | 0.51 | 0.80 (0.41, 1.56) | 0.51 |
| ER-/PR-/HER2- |  |  |  |  |  |
| Radon exposure <148 Bq/m^3^ | 284/2,409,280 | Referent |  | Referent |  |
| Radon exposure ≥148 Bq/m^3^ | 9/96,821 | 0.80 (0.41, 1.56) | 0.51 | 0.73 (0.37, 1.43) | 0.36 |

^a^ Adjusted for age, race.

^b^ Additionally adjusted for Census tract median home value, Census tract median income, marital status, living arrangements, individual-level income, region of residence, family history of breast cancer, screening mammography, personal history of biopsy-confirmed BBD, age at menarche, parity, age at first birth, lactation, hormone use (among postmenopausal women only), height, BMI at age 18, change in BMI since age 18, smoking status, physical activity, adult alcohol consumption, PM_2.5_ air pollution, population density.

**Table 6** Cumulative average radon and breast cancer risk stratified by residential mobility in NHSII

|  | Never moved | | Ever moved | |
| --- | --- | --- | --- | --- |
| Outcome^a^ | Cases/person-years | Fully adjusted^b^  HR (95% CI) | Cases/person-years | Fully adjusted^b^  HR (95% CI) |
| Invasive breast cancer |  |  |  |  |
| Radon quintile 1 | 135/82,695 | Referent | 668/413,799 | Referent |
| Radon quintile 2 | 173/96,293 | 1.11 (0.86, 1.44) | 637/407,664 | 1.00 (0.88, 1.12) |
| Radon quintile 3 | 189/104,868 | 1.10 (0.82, 1.46) | 608/398,254 | 0.99 (0.87, 1.13) |
| Radon quintile 4 | 184/126,512 | 0.85 (0.64, 1.14) | 561/372,837 | 0.99 (0.87, 1.14) |
| Radon quintile 5 | 208/121,752 | 1.02 (0.76, 1.36) | 603/378,021 | 1.06 (0.93, 1.22) |
| *p* for trend |  | 0.70 |  | 0.24 |
| Continuous radon (per IQR increase)^c^ | 889/532,121 | 1.01 (0.94, 1.07) | 3,077/1,970,574 | 1.00 (0.96, 1.04) |
| ER+ |  |  |  |  |
| Radon quintile 1 | 87/82,742 | Referent | 403/414,056 | Referent |
| Radon quintile 2 | 94/96,369 | 0.90 (0.64, 1.27) | 377/407,919 | 1.00 (0.86, 1.17) |
| Radon quintile 3 | 126/104,934 | 1.12 (0.78, 1.61) | 372/398,486 | 1.03 (0.87, 1.22) |
| Radon quintile 4 | 109/126,584 | 0.75 (0.52, 1.09) | 330/373,037 | 1.00 (0.84, 1.20) |
| Radon quintile 5 | 110/121,850 | 0.82 (0.56, 1.20) | 365/378,239 | 1.09 (0.91, 1.31) |
| *p* for trend |  | 0.14 |  | 0.26 |
| Continuous radon (per IQR increase)^c^ | 526/532,479 | 0.97 (0.89, 1.06) | 1,847/1,971,736 | 1.02 (0.96, 1.07) |
| ER- |  |  |  |  |
| Radon quintile 1 | 19/82,802 | Referent | 82/414,322 | Referent |
| Radon quintile 2 | 23/96,440 | 1.20 (0.61, 2.36) | 89/408,172 | 1.11 (0.80, 1.55) |
| Radon quintile 3 | 27/105,019 | 1.20 (0.57, 2.55) | 98/398,722 | 1.26 (0.89, 1.80) |
| Radon quintile 4 | 29/126,658 | 0.99 (0.46, 2.13) | 85/373,281 | 1.21 (0.84, 1.74) |
| Radon quintile 5 | 32/121,909 | 1.18 (0.55, 2.55) | 101/378,493 | 1.32 (0.91, 1.91) |
| *p* for trend |  | 0.85 |  | 0.22 |
| Continuous radon (per IQR increase)^c^ | 130/532,828 | 0.98 (0.82, 1.17) | 455/1,972,989 | 1.04 (0.94, 1.15) |
| ER+/PR+ |  |  |  |  |
| Radon quintile 1 | 69/82,758 | Referent | 348/414,101 | Referent |
| Radon quintile 2 | 84/96,380 | 1.04 (0.72, 1.50) | 336/407,951 | 1.04 (0.88, 1.22) |
| Radon quintile 3 | 114/104,943 | 1.30 (0.88, 1.93) | 327/398,526 | 1.07 (0.89, 1.28) |
| Radon quintile 4 | 95/126,600 | 0.85 (0.56, 1.28) | 289/373,075 | 1.04 (0.86, 1.25) |
| Radon quintile 5 | 93/121,862 | 0.91 (0.60, 1.38) | 319/378,287 | 1.13 (0.94, 1.37) |
| *p* for trend |  | 0.14 |  | 0.21 |
| Continuous radon (per IQR increase)^c^ | 455/532,542 | 0.98 (0.89, 1.07) | 1,619/1,971,941 | 1.02 (0.97, 1.08) |
| ER-/PR- |  |  |  |  |
| Radon quintile 1 | 16/82,804 | Referent | 73/414,333 | Referent |
| Radon quintile 2 | 16/96,448 | 1.06 (0.49, 2.28) | 77/408,186 | 1.06 (0.75, 1.51) |
| Radon quintile 3 | 23/105,023 | 1.45 (0.63, 3.32) | 81/398,740 | 1.12 (0.76, 1.63) |
| Radon quintile 4 | 26/126,660 | 1.29 (0.55, 3.00) | 78/373,286 | 1.18 (0.80, 1.73) |
| Radon quintile 5 | 31/121,910 | 1.59 (0.68, 3.71) | 92/378,499 | 1.27 (0.86, 1.88) |
| *p* for trend |  | 0.28 |  | 0.18 |
| Continuous radon (per IQR increase)^c^ | 112/532,845 | 1.04 (0.86, 1.25) | 401/1,973,044 | 1.04 (0.94, 1.16) |
| ER+/PR- |  |  |  |  |
| Radon quintile 1 | 18/82,808 | Referent | 52/414,359 | Referent |
| Radon quintile 2 | 9/96,454 | 0.35 (0.13, 0.89) | 38/408,225 | 0.71 (0.45, 1.13) |
| Radon quintile 3 | 11/105,037 | 0.35 (0.13, 0.97) | 42/398,782 | 0.76 (0.46, 1.25) |
| Radon quintile 4 | 14/126,670 | 0.38 (0.14, 1.00) | 39/373,314 | 0.77 (0.46, 1.28) |
| Radon quintile 5 | 17/121,929 | 0.44 (0.17, 1.18) | 45/378,529 | 0.83 (0.49, 1.38) |
| *p* for trend |  | 0.85 |  | 0.97 |
| Continuous radon (per IQR increase)^c^ | 69/532,896 | 0.93 (0.71, 1.21) | 216/1,973,209 | 0.99 (0.85, 1.15) |
| ER-/PR-/HER2- |  |  |  |  |
| Radon quintile 1 | 9/82,813 | Referent | 40/414,360 | Referent |
| Radon quintile 2 | 10/96,455 | 1.22 (0.45, 3.32) | 43/408,221 | 1.04 (0.65, 1.67) |
| Radon quintile 3 | 7/105,039 | 0.68 (0.20, 2.30) | 45/398,781 | 1.09 (0.66, 1.82) |
| Radon quintile 4 | 17/126,667 | 1.33 (0.42, 4.18) | 44/373,313 | 1.19 (0.71, 1.99) |
| Radon quintile 5 | 19/121,923 | 1.40 (0.44, 4.46) | 59/378,529 | 1.41 (0.84, 2.35) |
| *p* for trend |  | 0.35 |  | 0.11 |
| Continuous radon (per IQR increase)^c^ | 62/532,897 | 1.04 (0.81, 1.34) | 231/1,973,204 | 1.08 (0.95, 1.24) |

^a^ Radon quintile 1: <27.0 Bq/m^3^; quintile 2: ≥27.0-37.7 Bq/m^3^; quintile 3: ≥37.7-50.1 Bq/m^3^; quintile 4: ≥50.1-74.9 Bq/m^3^; quintile 5: ≥74.9 Bq/m^3^.

^b^ Adjusted for age, race, Census tract median home value, Census tract median income, marital status, living arrangements, individual-level income, region of residence, family history of breast cancer, screening mammography, personal history of biopsy-confirmed BBD, age at menarche, parity, age at first birth, lactation, menopausal status and hormone use (among postmenopausal women only), height, BMI at age 18, change in BMI since age 18, smoking status, physical activity, adult alcohol consumption, PM_2.5_ air pollution, population density.

^c^ An IQR increase in cumulative average radon is 37.3 Bq/m^3^.
